# Placental gene expression and antibody levels of mother-neonate pairs reveal an enhanced risk for inflammation in a helminth endemic country

**DOI:** 10.1038/s41598-019-52074-z

**Published:** 2019-10-31

**Authors:** Esther Ludwig, Jutta Harder, Matthew Lacorcia, Yabo Josiane Honkpehedji, Odilon Paterne Nouatin, Govert J. van Dam, Paul L. A. M. Corstjens, Erliyani Sartono, Meral Esen, Silvia M. Lobmaier, Ayola Akim Adegnika, Clarissa Prazeres da Costa

**Affiliations:** 1 Institute for Medical Microbiology, Immunology and Hygiene, Technische Universität München, Munich, Germany; 2 Centre de Recherches Médicales de Lambaréné, Lambaréné, Gabon; 3 Department of Parasitology, Leiden University Medical Centre, Leiden, The Netherlands; 4 Institut für Tropenmedizin, Universität Tübingen, Tuebingen, Germany; 5 Frauenklinik und Poliklinik, Klinikum rechts der Isar, Technische Universität München, Munich, Germany; 6 German Centre for Infection Research, Tuebingen, Germany; 7 Department of Cell and Chemical Biology, Leiden University Medical Center, Leiden, The Netherlands

**Keywords:** Epidemiology, Genetics research

## Abstract

*In utero* exposure to environmental factors can modify the development of allergies later in life whereby the mechanisms of the feto-maternal crosstalk still remain largely unknown. Murine studies revealed that inflammatory maternal signals elicited by chronic helminth infection within the placenta imprint a distinct gene expression profile related to the Vitamin-D-receptor (VDR)-inflammation-axis. We thus investigated whether pro- or anti- inflammatory immune responses as well as VDR and related gene expression within the placenta differ between women from helminth-endemic and non-endemic areas. A prospective pilot study was conducted in Munich, Germany (helminth non-endemic) and Lambaréné, Gabon (helminth-endemic). At delivery, clinical information alongside placenta tissue samples and maternal and cord blood were obtained for further laboratory analysis. *Schistosoma haematobium* infection was detected in 13/54 (23%) Gabonese women. RT PCR revealed significantly lower gene expression of VDR, Cyp27b1, Foxp3 and *IL10* in Gabonese compared to German placentae as well as significantly lower levels of plasma IgG4 in newborns resulting in a significantly higher IgE/IgG4 ratio. These findings demonstrate that exposure *in utero* to different environments alters placental gene expression and thus possibly plays a role in the development and modulation of the immune system of the offspring.

## Introduction

Chronic inflammatory disorders, such as allergic airway inflammation, are negatively associated with high levels of helminth infection such as schistosomes, particularly in low-income countries^1^ where prevalence of allergic reactivity and autoimmune antibodies increases after anthelmintic treatment^2–4^. Approximately 220 million people are infected with schistosomes^5^ and thus, approximately 40 million women of childbearing age. Yet little is known about the specific morbidities inflicted on pregnant women and their offspring^6^. Complications such as anaemia and low-birth-weight^7^, as well as skewed immune responses, which can alter susceptibility for other diseases^8^, are just some examples of the potential impact of a pregnancy modulated by helminth infection. Such infections are known to modify host immune responses through a variety of mechanisms^9^, minimizing host-damage to enable survival of the parasite^10^. Stable, chronic helminth infections can endure for long periods (up to 20 years) in an individual host, facilitated through helminth-induced recalibration of host immunity towards a state of immune hyporesponsiveness that can be considered a form of immunologic tolerance^11–13^.

Recent research indicates that early exposure to such a modified environment during gestation already primes the fetal immune system *in utero* and leads to enhanced immunological maturity at birth^14^. Indeed, it was recently demonstrated, that experimental chronic infection with the helminth *Schistosoma mansoni* during pregnancy influences the outcome of allergic asthma in offspring^15^. This was further associated with downregulation of genes associated with either Vitamin-D-metabolism and –pathways such as the transcription factor Vitamin-D-receptor (VDR)^16^, the enzyme 1α-hydroxylase (Cyp27b1), responsible for vitamin D activation, as well as hydroxy-delta-5-steroid dehydrogenase (Hsd3b1)^15^, which is crucial for roles in the biosynthesis of all hormonal steroids^17^.

Therefore, defective VDR signalling in placental tissue might result in increased risk of placental inflammation and expression of inflammatory cytokines or dampening of anti-inflammatory and tolerogenic cytokines, respectively. Placental VDR expression, as well as Cyp27b1, has also been linked to regulation of key cytokines involved in inflammatory responses, namely interleukin 10 (IL-10) or interferon gamma (IFN-γ). Cyp27b1 hydroxylases’ 25(OH)D to the active form 1,25 (OH)_2_D, whereas Cyp24a1 is responsible for the inactivation. VDR and Cyp27b1 are expressed in almost all immune cells as well as in both decidua^18^ and trophoblast^19^ suggesting that the placenta itself converts 25(OH)D to the active form and may thereby function in an autocrine or paracrine fashion^20^. Indeed, the loss of 1,25 (OH)_2_D production in the fetal compartment of the placenta has been shown to cause generalized dysregulation of placental inflammation after immune challenge^20^, which are known to be induced during helminth infection^21,22^. IL-10 is produced by Forkhead-Box-Protein P3 (Foxp3) expressing regulatory T cells alongside other cell types such as the villous cytotrophoblasts within the placenta, where it appears to work as a key facilitator of successful pregnancy^20^.

The placenta plays a decisive role in pregnancy maintenance and the development and protection of the fetus. Besides the production of hormones, the placenta is a barrier between mother and fetus and maintains immunological tolerance. The mature, disc-shaped placenta can be divided into three zones. First the basal plate or decidua which is predominantly the maternal side of the placenta and consists of up to 30–40% leukocytes^23^ to avoid rejection of the fetus as well to protect it from maternal infections. The placental leukocyte population is compromised by around 70% uterine natural killer cells (uNKs), about 20% macrophages and 10% T-lymphocytes (with 10–15% regulatory T-cells), but also dendritic cells and mast cells can be found in the early placental bed^24,25^. The fetal side is composed of the chorionic plate. The feto-maternal zone in-between consists in the intervillous space with maternal blood and the villous trees providing the fetal blood.

Environmental triggers and maternal stress can lead to significant changes within to the placenta, with important outcomes for fetal health and development^26^. In multivariate models adjusted for geohelminths, maternal schistosomiasis was associated with increased levels of inflammatory cytokines in maternal peripheral blood, placental, and cord blood, as well as acute subchorionitis^27^. Granulomatous inflammation in the placenta^28^ and the cervix^29^ in the context of female genital schistosomiasis (FGS) can occur if immature worms or eggs directly become lodged in the placenta^6^. In general, placental inflammation is associated with substantially lower feto-maternal immunoglobin G (IgG) antibody transfer efficiency^30^ which is tightly regulated and mediated by neonatal Fc Receptor (FcRn)^31^. IgG is the only antibody subclass which is able to cross the placental barrier. IgG, especially its subclasse like IgG4, and IgE, which acts in a competitive way with IgG4, are strongly induced during helminth infections^32^. Strong anti-parasite IgE responses are associated with resistance to infection^33^, whereas high levels of IgG4 have been associated with susceptibility^34^. However, in contrast to IgG4, IgE is not able to cross the placental barrier. Thus, any detection of fetal IgE can be considered as evidence for *in utero* priming of the fetal immune system. Indeed, schistosome specific IgE have been detected in cord blood from *S. haematobium* infected mothers^35^.

Considering that the placental anti- as well as proinflammatory gene expression can be skewed by the environment including helminth infections, we compared placental gene expression as well as inflammation markers in maternal and cord blood of Germans and Gabonese cohorts in a cross-sectional study.

## Results

### Differences in maternal and neonatal characteristics between gabon and germany

For the study, 54 pregnant women in Gabon, and 47 in Germany fulfilled all inclusion criteria and were successfully included into the study (Table 1). At birth we registered data related to maternal health, pregnancy, birth outcome, and neonatal health as shown in Table 1. Significant differences between the two study populations emerged not only between the mothers but also between the newborns. Gabonese mothers were on average seven years younger than the mothers in the German control cohort. In both cohorts, the mothers delivered their newborns on average at 39 weeks of gestation and had the same number of deliveries, whereas the higher number of gravidities in Gabon indicates a higher rate of pregnancy loss (naturally and spontaneously) than in Germany. Furthermore, women in Gabon had a reduced haemoglobin (10.6 ± 2.21 vs. 12.15 ± 1.11 g/dl) rendering most women anaemic. CRP values at delivery were elevated in Gabonese women (1.09 ± [0.63;2.17] vs. 0.63 ± [0.24;1.13] mg/dl). Even though sampling was performed during the summertime in Germany, 25-OH-Vitamin D plasma concentration was significantly higher in Gabonese maternal peripheral blood samples (33.4 ± 8.7 ng/mL vs. 23.9 ± 13.8 ng/mL) as well as in Gabonese cord blood samples (36.0 ± 8.64 ng/mL vs 28.94 ± 15.66 ng/ml) (Table 1). The month of sample collection (Supplementary Fig. 2) in both countries nor infection status in the Gabonese cohort had an influence on 25-OH-Vitamin D concentrations in Gabon (Supplementary Fig. 2). No difference in gender distribution of neonates between both countries was seen. Gabonese newborns were, however, significantly shorter (3 cm) and on average 255 g lighter than their German counterparts.

**Table 1:** Maternal and newborn characteristics at delivery in Gabon and Germany.

Number n	Gabon	Germany	p value
	54	47	
**Mother**			
Age [years]	26 ± 7	33 ± 5	**<0.0001**
Parity	2 ± 2	1.7 ± 0.7	0.51
Gravidity	4 ± 2	2.1 ± 1.5	**<0.0001**
Gestational age at delivery (weeks)	39.3 ± 2	39.7 ± 1.2	**0.20**
Haemoglobin [g/dL]	10.6 ± 2.21	12.15 ± 1.11	**0.0011**
White blood cells [10^3/mm^3]	11.81 ± 5.15	13.11 ± 3.82	0.26
Eosinophils [%]	1.91 ± 1.91	Not done	
25-OHD-Vitamin D3 concentration [ng/mL]	34.5 ± 8.8	23.9 ± 13.8	**<0.0001**
Calcium [mmol/L]	2.29 ± 0.12	2.31 ± 0.11	0.42
CRP [mg/dL]	1.09 ± [0.63;2.17]	0.63 ± [0.24;1.13]	**0.0022**
**Newborn**			
Gender (male)	30 (56)	22 (47)	0.38
Length [cm]	50 ± 2	53 ± 3	**<0.0001**
Birthweight [g]	3076 ± 510	3331 ± 544	**0.0012**
25-OHD-Vitamin D3 concentration [ng/mL]	36.0 ± 8.64	28.94 ± 15.66	**0.0011**
Calcium [mmol/L]	2.66 ± 0.22	2.79 ± 0.15	**0.0004**
CRP [mg/dL]	0.01 ± [0.01;0.02]	0.01 ± [0.01;0.02]	0.56

Data are presented as mean ± SD (n), median with IQR (n) for C-reactive Protein (CRP), values or as numbers (%) where indicated; T-test was performed where data are normal distributed; for data without normal distribution a two-tailed Mann Whitney U-test was performed; blood for plasma parameters was taken from maternal peripheral vein blood or from cord blood, respectively.

Comparing the Gabonese cohort, with participants with and without *S. haematobium* infection, none of the mentioned outcome parameters differed significantly (Supplementary Table 1).

### Lower relative gene expression of steroid and vitamin D pathway associated genes in gabonese placenta tissue

**Figure 1:**
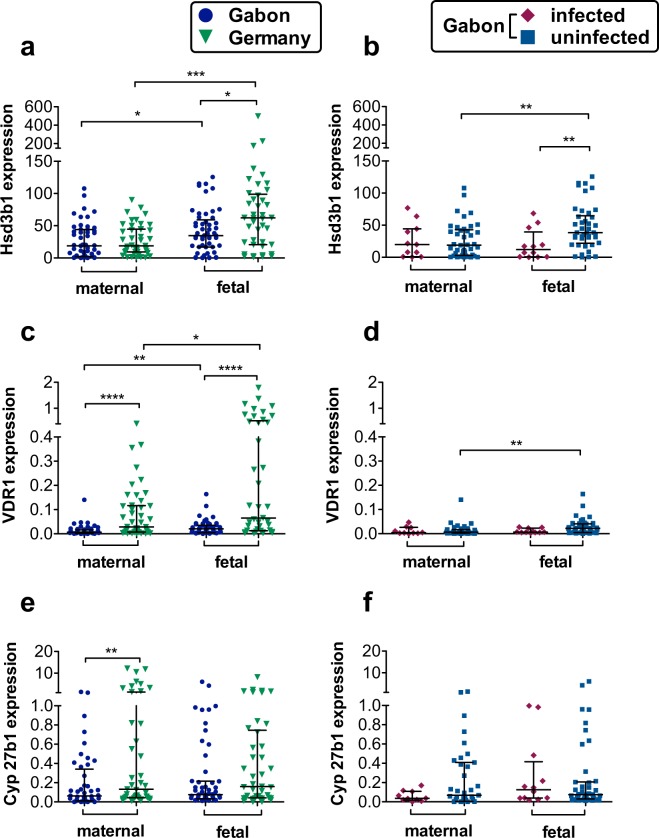
Steroid and Vitamin D pathway associated gene expression within the maternal and fetal side of the placenta. Relative Hsd3b1, VDR and Cyp27b1 expression, normalized to hypoxanthine-guanine phosphoribosyl transferase (HPRT) as house-keeping gene (HKG). All data are shown with median and interquartile range. P values are for Mann-Whitney U-tests. Comparison between the two maternal, or fetal groups, respectively. P value: *< 0,05; **< 0,01; ***< 0,001; ****< 0,0001; n (maternal, Gabon) = 50; n (maternal, Germany) = 44; n (fetal, Gabon) = 53; n (fetal, Germany) = 41; n (Gabon, maternal, uninfected) = 40; n (Gabon, maternal, infected) = 10 n (Gabon, fetal, uninfected) = 42; n (Gabon, fetal, infected) = 11. **(a)** Expression of Hsd3b1 in Gabonese and German placenta tissue. **(b)** Hsd3b1 gene expression in placentae from infected or uninfected Gabonese mothers. **(c)** Expression of VDR1 in Gabonese and German placenta tissue. **(d)** VDR1 gene expression in placentae from infected or uninfected Gabonese mothers. **(e)** Expression of Cyp27b1 in Gabonese and German placenta tissue. **(f)** Cyp27b1 gene expression in placentae from infected or uninfected Gabonese mothers.

In order to study the potential effects of distinct geographical environments upon placental inflammation, in association with the effects of helminth infection, we compared the placental expression levels of genes known to be important for proper placental function through steroid and Vitamin D pathways between the Gabonese and German mothers. As shown in Fig. 1(a–f), comparative analyses of gene expression between Gabonese and German placenta samples revealed a significantly lower expression for VDR1 and Cyp27b1 on the Gabonese maternal placental side, whereas no differences were detected regarding Hsd3b1 expression. Regarding the fetal side of the placenta of the total Gabonese and German cohort, significantly lower expression for Hsd3b1 and VDR1 was found in the Gabonese when compared to the German samples and no differences in expression of Cyp27b1. However, these genes were expressed to a greater extent when compared to their corresponding maternal side (Fig. 1a,c). Within the Gabonese cohort, we compared placenta samples of mothers infected with *S. haematobium* to those uninfected Gabonese mothers (Fig. 1b,d,f), and showed a lower expression of Hsd3b1 on the fetal side of placenta from infected mothers. No differences for the VDR1 and Cyp27b1 expression between the infected and uninfected cohort was detected.

### Lower relative gene expression of Foxp3 and IL10 in Gabonese placental samples

**Figure 2:**
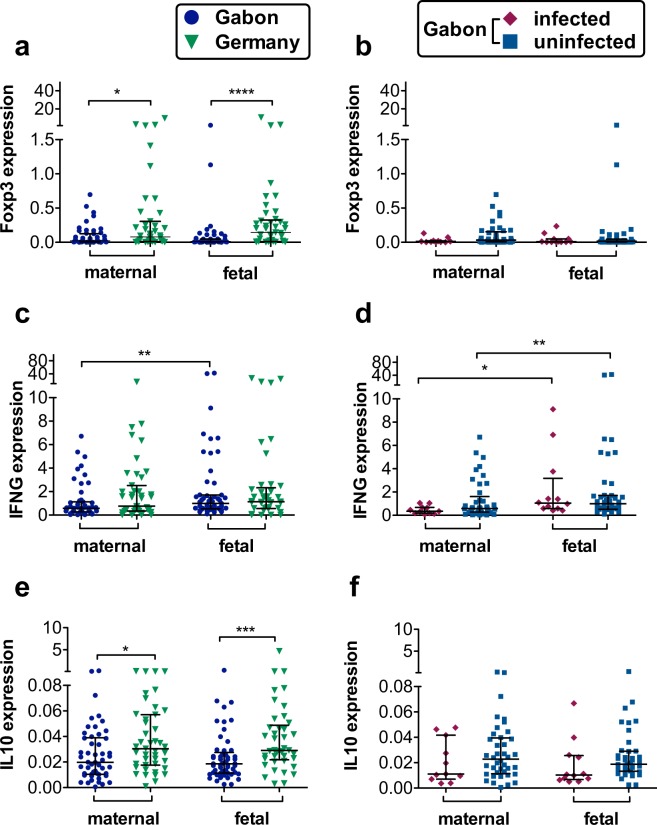
Expression of immunologically relevant genes within the maternal and fetal side of the placenta. Relative Foxp3, *IFNG*, and *IL10* expression, normalized to HPRT. All data are shown with median and interquartile range. P values are for Mann-Whitney U-tests. Comparison between the two maternal, or fetal groups, respectively. P value: *< 0,05; **< 0,01; ***< 0,001; ****< 0,0001; n (maternal, Gabon) = 50; n (maternal, Germany) = 44; n (fetal, Gabon) = 53; n (fetal, Germany) = 41; n (Gabon, maternal, uninfected) = 40; n (Gabon, maternal, infected) = 10 n (Gabon, fetal, uninfected) = 42; n (Gabon, fetal, infected) = 11; **(a)** expression of Foxp3 in Gabonese and German placenta tissue. **(b)** Foxp3 gene expression in placentae from infected or uninfected Gabonese mothers. **(c)** expression of *IFNG* in Gabonese and German placenta tissue **(d)** *IFNG* gene expression in placentae from infected or uninfected Gabonese mothers. **(e)** expression of *IL10* in Gabonese and German placenta tissue. **(f)** *IL10* gene expression in placentae from infected or uninfected Gabonese mothers.

Dynamic processes of tolerance and regulation of inflammatory signals take place within the placenta. As such, the expression of immune-related genes known to be expressed in the placenta, such as Foxp3, *IFNG* or *IL10*, was compared between the Gabonese and German samples (Fig. 2a,c,e). There was lower expression of Foxp3 and *IL10* on the maternal side of the placenta in Gabonese samples compared to those from the German cohort (Fig. 2a,e), whereas *IFNG* was equally expressed in Gabonese and German placentae. Foxp3 and *IL10* also exhibited significantly lower relative expression on fetal side of Gabonese placental samples compared to those from the German cohort (Fig. 2a,e). Comparing placental gene expression between infected and uninfected Gabonese mothers as shown in Fig. 2b,d,f showed no difference between the two cohorts. *IFNG* was lower expressed on the maternal side. Infection status within the Gabonese cohort had no influence on the gene expression (Fig. 2b,d,f).

### IgG4 correlation between Gabonese and German mother-child pairs and significantly higher schistosome-specific IgG4 in mother-child plasma samples from infected Gabonese mothers

**Figure 3:**
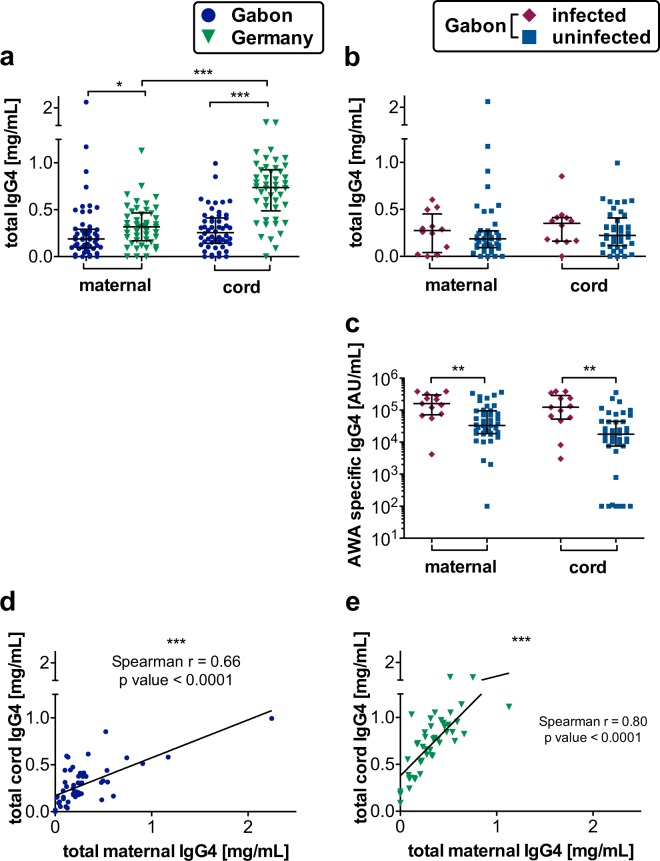
Maternal and fetal plasma IgG4 levels and their correlation in Gabon and Germany. IgG4 levels were measured in plasma via ELISA. All data are shown with median and interquartile range. P values are for Mann-Whitney U-tests or Spearman’s rank correlation, respectively. Comparison between the two maternal, or cord blood groups, respectively. In the case additionally comparison between maternal and cord blood group each to show the placental transfer; P value: *< 0,05; **< 0,01; ***< 0,001; ****< 0,0001; n (maternal, Gabon) = 54; n (maternal, Germany) = 47; n (cord, Gabon) = 53; n (cord, Germany) = 47; n (Gabon, maternal, uninfected) = 42; n (Gabon, maternal, infected) = 12 n (Gabon, cord, uninfected) = 42; n (Gabon, cord, infected) = 12; **(a)** total IgG4 levels in all German maternal all Gabonese plasma samples **(b)** total IgG4 levels in Gabonese plasma samples divided into infected and uninfected group. **(c)** AWA specific IG4 levels in in Gabonese plasma samples divided into infected and uninfected group. **(d)** Correlation between general maternal and cord blood IgG4 levels in Gabonese plasma samples; number of pairs = 53; spearman r = 0,66, p value = < 0,0001. (**e**) Correlation between general maternal and cord blood IgG4 levels in German plasma samples; number of pairs = 45; spearman r = 0.80; p value = < 0,0001.

IgG4 plays an important role during helminth infections and its placental transfer can be diminished by placental inflammation. In German maternal and cord blood plasma samples, the total IgG4 levels were significantly higher compared to those from Gabon. However, the increase in fetal IgG4 which we observed in German cord blood, was not detectable in Gabonese samples, indicating that the active antibody-transport was diminished in the Gabonese placentas irrespective to their infection status (Fig. 3a,b). In the Gabonese cohort infection status had no influence on total IgG4 levels, but indeed on AWA specific IgG4 levels, which were significantly higher in maternal and cord blood in the infected group. Nevertheless, non-infected mothers still had substantial background antibodies possibly due to previous exposure to schistosomiasis or other helminths (Fig. 3c). Additionally, there is a strong correlation between maternal and cord blood total IgG4 concentrations in all groups (Fig. 3d,e) however stronger in the German cohort.

### Significantly higher total and AWA specific IgE levels and IgE/IgG4 ratio in Gabonese plasma samples

**Figure 4:**
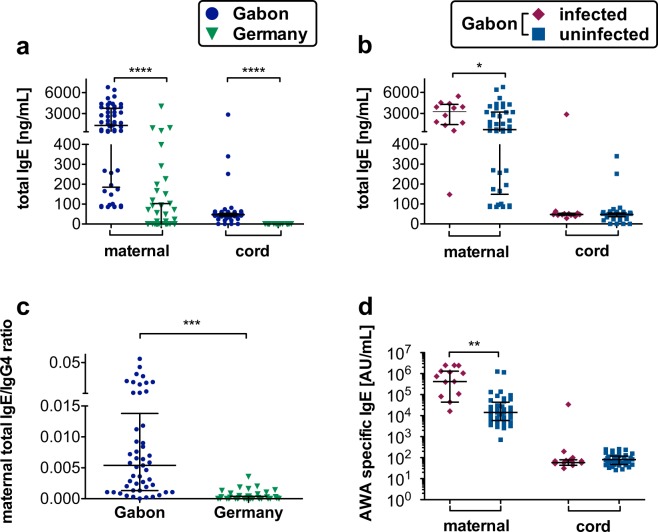
Maternal and fetal plasma IgE levels and the IgE/G ratio in Gabon and Germany. IgE levels were measured in plasma via ELISA. All data are shown with median and interquartile range. P values are for Mann-Whitney U-tests. Comparison between the two maternal, or cord blood groups, respectively. P value: *< 0,05; **< 0,01; ***< 0,001; ****< 0,0001; n (maternal, Gabon) = 54; n (maternal, Germany) = 47; n (cord, Gabon) = 53; n (cord, Germany) = 47; n (Gabon, maternal, uninfected) = 42; n (Gabon, maternal, infected) = 12 n (Gabon, cord, uninfected) = 42; n (Gabon, cord, infected) = 12; **(a)** Total IgE levels in all German maternal all Gabonese plasma samples **(b)** Total IgE levels in Gabonese plasma samples divided into infected and uninfected group. **(c)** IgE/IgG4 ratio in maternal plasma samples. **(d)** AWA specific IgE levels in Gabonese plasma samples divided into infected and uninfected group.

Large amounts of specific and nonspecific IgE are a well-recognized feature of the immune response to parasitic helminth infections, including schistosomiasis. IgG4 and IgE influence susceptibility or protection against helminth infections. Comparing total IgE levels in maternal and cord blood plasma between Gabon and Germany yielded significant differences with strongly elevated levels in Gabonese samples which were further increased in the group of mothers infected with *S. haematobium* (Fig. 4a,b). Interestingly and again irrespective of the maternal infection status we detected IgE in 51 of 53 Gabonese cord blood samples but not in any German cord blood samples (Fig. 4a,b). AWA specific IgE levels were increased in maternal but not fetal plasma from infected Gabonese mothers (Fig. 4d). The ratio of total maternal IgE/IgG4 was significantly higher in Gabonese samples than in German samples (Fig. 4c).

## Discussion

*In utero* exposure to helminths and other environmental factors has recently been shown to alter placental gene expression^36^ and to influence the offspring’s propensity to develop allergies later in life^14,15^. Previous investigations of gene expression within the placental tissue have rarely considered potential expression differences between the fetal and maternal side of the placenta or possible differences in environmental exposure^37,38^. Indeed, Hsd3b1, the only isoform expressed in the placenta, and required for the biosynthesis of progesterone as well as for the maintenance of pregnancy and fetal growth^17,39^, was expressed to a significantly lower level within the fetal side of Gabonese placentas, and even lower within the sub-group from *S. haematobium* infected mothers. This might be connected to the higher number of previous pregnancy losses as well as lower birth weight and size of the children in this cohort. Furthermore, progesterone functions as an anti-inflammatory hormone and a decreased expression might thereby contribute to placental inflammation. In addition, the reduced expression of the Vitamin-D related genes VDR and Cyp27b1 on the maternal side of the Gabonese placenta samples alongside the overall differences in gene expression levels between the maternal and fetal side are indicative for independent regulation across these distinct tissue niches. Due to VDR’s important role as transcription factor and its presence in almost all kinds of immune cells, the reduction of VDR and Cyp27b1 as well as Foxp3 and *IL10* expression in the Gabonese placentas could indicate that Gabonese mothers are more prone to host an inflammatory immune milieu in the placenta. The proinflammatory cytokine IFN-γ is present mainly in the early placenta and gestational endometrium and at later stages the trophoblasts dampen IFN-γ signalling strongly^40^ to avoid rejection of the fetus and possibly strong infection-mediated inflammation. No differences in placental *IFNG* gene expression, as one hallmark of inflammation, was found between both groups, although reports indicate that trophoblast cells only respond selectively to IFN-γ^40^ so rather pregnancy and related hormones than maternal infection influence its expression. In this study, elevated CRP levels in most women of the Gabonese cohort hinted towards low-grade inflammation. Except for Hsd3b1, infection with *S. haematobium* did not have an influence on Vitamin D pathway associated (VDR, Cyp27b1) or immunological (*IL10, IFNG*, Foxp3) genes as well as vitamin D plasma levels. Vitamin D has recently been shown to induce the antimicrobial peptide LL-37 which belongs to the cathelicidin family^41–44^ and has a potent antimicrobial activity against a wide assortment of bacterial, mycobacterial, protozoal, fungal and viral pathogens. Yet, until now, nothing is known about its role in helminthic diseases such as schistosomiasis. Within this study however, the schistosomiasis-infected group was too small to allow assessment of this parameter but will be included in future studies. In conclusion, environmental and perhaps genetic factors might suppress anti-inflammatory signals and alter the regulation and activation of vitamin D and the regulation of tolerogenic and anti-inflammatory markers.

Helminths provoke a subtle inflammation in their hosts and cause anaemia, also known as anaemia of infection or of chronic disease^45–48^. The main cause is proinflammatory cytokines such as TNF-α or IL-6^6^. The women from Gabon, which is a highly endemic area for schistosomiasis^49^, indeed suffered from anaemia in contrast to the European women even though lower haemoglobin concentrations during pregnancy are physiologic (cut-off for pregnant women: haemoglobin <11 g/dL). This confirmed findings from another study with 998 pregnant women in Gabon, where 49.9% of the participants were anaemic^7^. Anaemia however is most likely to have multifactorial causes in this geographical region which besides schistosomiasis and pregnancy itself includes malaria, soil transmitted helminths and nutritional deficiencies such as iron deficiency.

Placental inflammation and pro-inflammatory cytokines might also contribute to fetal hypoxia^6,45^ and adverse birth outcomes, such as low birth weight^7^. Indeed, neonates in Gabon were significantly smaller (minus 3 cm) and lighter (minus 255 g) than those born in Germany. In addition, young maternal age has been shown to be associated to low birth weight, too^50^ and mothers in Gabon are younger than the Germans, but this might be an additional factor beside geographical difference.

Another consequence of placental inflammation is disorder of the placental barrier with a disruption of the active Fc receptor mediated antibody transfer. Cord blood IgG4 compared to maternal levels was significantly higher in Germany, whereas Gabonese cord and maternal IgG4 levels did not differ from each other. As this isotype represents a maternally-derived antibody, these results indicate disturbances to the processes involved in the transfer from maternal circulation in Gabon. Because of the small number of schistosome infected mothers (23%, n = 13) in our study, the noted differences are more likely to be influenced by other factors than acute schistosomiasis. Six Gabonese women (11%) stated to have had malaria during pregnancy whereas at time of delivery only one Gabonese mother had *Plasmodium falciparum* in her thick blood smear and was thus excluded from this study. Malaria could contribute to disturbed placental transfer taking into consideration that infections may be asymptomatic in high endemic areas for malaria and infections therefore obscure^49^.

Overall levels of IgG4 were significantly lower in Gabonese plasma, although the infected sub-group showed a trend towards higher IgG4. AWA-specific IgG4 levels were significantly higher in cord and maternal blood of the schistosome-infected group as expected in a population endemic for helminth infections since there exists substantial cross reactivity between helminth antigens^32^. IgG4 predominantly supports an immune-tolerant environment for helminths by counteracting helminth-specific IgE responses. High IgE levels are associated with resistance to infection^33^, whereas high levels of IgG4 have been associated with susceptibility^34^. In our study population total IgE levels were significantly higher in the Gabonese cohort and within this cohort also higher in the maternal plasma samples of the infected group. The high levels in some maternal German plasma samples were correlated to their history of allergy since severe autoimmune diseases were part of the exclusion criteria and therefore excluded. Regarding interactions between IgE and IgG4, a high IgE/IgG4 ratio correlates with protection against helminth infections. Indeed, in Gabon the maternal total IgE/IgG4 ratio is significantly higher than in Germany and lifelong exposures might induce protection against reinfection.

In contrast to IgG4, IgE cannot cross the placental barrier thus the IgE antibodies detected in cord blood are most likely already produced by the fetus *in utero*. Unlike the German cohort, about 98% Gabonese cord blood samples had detectable levels of IgE antibodies. In a study conducted in Coast Province, Kenya, cord blood lymphocytes were stimulated *in vitro* and about 36% of healthy newborns spontaneously produced polyclonal IgE and IgG^51^. Polyclonal and parasite-specific IgE were equally present in Kenyan cord and maternal sera whilst it remained undetectable in cord blood sera from North American infants^52^. Likewise, in Gabonese cord blood *Schistosoma-*specific IgE antibodies were detected previously^35^. However, in this study, even though levels of AWA-specific IgE were high in maternal blood, none could be detected in the cord blood. An explanation could be adult worm antigens may not cross the placenta in the same quantity as egg-related antigens^35^. The immune reaction to AWA is less strong than to the eggs and SEA^53,54^. Worms are able to survive for many years in the human host due to their ability to mask their surface tegument with host antigens^55^ and the excretion of an only small amount of antigenic molecules.

In summary, our results indicate that pregnant women in Gabon might have a higher risk for placental inflammation, with a lower anti-inflammatory milieu when compared to German women. This is associated with reduced transplacental IgG4 transfer whereby the detection of IgE in offspring could be a hint for early priming events such as the de novo antibody class-switching due to *in utero* exposure to (helminthic) antigens. Furthermore, as one of the first studies to compare inflammatory genes between geographically distinct populations, including subgroups based on helminth infection, as well as localized gene expression to placental and fetal placental subregions, we provide an initial setup for further investigation into how infection status can modify the complex crosstalk between placental inflammatory responses and healthy fetal development.

### Limitations

The neonates in Gabon and Germany were examined by different midwives. Except for Vitamin D, Ca++ and CRP levels, maternal hemogram was done in two different laboratories for each cohort and helminth diagnostic only for the Gabonese cohort, whereby we attempted to reduce the probability of infection to a maximum by using a questionnaire in the German group. Only 30 Gabonese women provided stool samples to investigate for geo-helminth infections.

The placenta samples were not washed, and gene expression analysis might be influenced by the presence of blood cells.

## Materials and Methods

### Study population

The study was approved by the Institutional ethic committee of CERMEL in BP 242 Lambaréné, Gabon, (CEI-007/2017) and the Ethikkommission der Fakultät für Medizin der Technischen Universität München, Ismaninger Straße 22, 81675 München, Munich, Germany, (22.11.2013, project number 385/13). It was financed by Deutsche Forschungsgemeinschaft DFG (CO 1469/14–1) and Deutsches Zentrum für Infektionsforschung (DZIF) e. V. (TI 07.003). The methods were carried out in accordance with the relevant guidelines and regulations. The study took place at two sites. First between June and August 2015 and Mai and August 2017 at the Klinikum rechts der Isar, Munich, Germany, where 57 pregnant women were screened and samples of 47 participants as European non-endemic control group could be collected and be included for analysis. All mothers were Caucasians. As the samples were all collected in Germany, we call them “German samples”. The second population consisted of 59 women living in the province of Moyen-Ogooue, Gabon, in central Africa and delivering their newborns between August and November 2017 either at the Albert Schweitzer Hospital or the Centre Hospitalier Rérgional Georges Rawiri (CHRGR) in Lambaréné. 54 women and their newborn fulfilled inclusion criteria. We call these samples “Gabonese samples”. The purpose of the study and the procedures involved were explained and only those mothers granting written informed consent were enrolled as participants. To participate, all adolescents were at least 18 years old, had ≥ 37 weeks of gestation, and were giving spontaneous vaginal birth. Caesarean section, as well as acute severe infections such as malaria or knowing of other chronic diseases such as hepatitis and HIV at time of delivery were exclusion criteria.

### Sample collection

Paired umbilical cord and maternal peripheral venous blood samples were collected at delivery. To avoid admixture of maternal and cord blood, cord blood was obtained not by squeezing the umbilical cord but instead by direct needle aspiration, taking care to clean the cord of maternal blood beforehand. All blood samples were collected in 9 ml NH_4_-Heparin (BD Vacutainer S-Monovette®) tubes for further peripheral blood mononuclear cell- (PBMC) and cord blood mononuclear cell- (CBMC) processing and for plasma separation. Plasma (2,5 mL) was separated by centrifugation and frozen at – 20 °C. Moreover, for the Gabonese cohort, EDTA blood for parasitology diagnostic and for clinical analyses was taken at the same time point and immediately analysed for the presence of protozoan blood parasites.

Placenta tissue from both, maternal and fetal side, was collected. For the maternal side we took about 0,5 cm^3^ directly from a macroscopic not calcified region in the middle of the placenta. For the fetal samples, we prepared the chorionic plate away to take a 0,5 cm^3^ big sample from the intervillous space. The tissue was put into labelled cryotubes containing 700 μl RNAlater® solution to stabilize placental RNA. The samples were stored at 4 °C for 24 to 48 hours and then transferred to −20° for long time storage and shipment on dry ice to Munich, Germany.

### Parasitological examination

These were performed in Gabonese specimens only. Thick blood smears of maternal peripheral blood were stained with Giemsa and examined microscopically for plasmodia and filaria. The mothers provided at least one urine sample for *S. haematobium* egg-count by microscopy; where possible the samples were frozen at −20 °C for shipment and further analysis. Furthermore, levels of schistosome circulating antigen (circulating anodic antigen [CAA]) were measured in cord and maternal peripheral plasma as well as in urine utilizing an immunochromatography based assay^56^.This lateral flow (LF) test applies luminescent upconverting particles (UCP) to quantitatively measure CAA levels with a 10 pg/mL lower limit threshold, analysing respectively 20 or 10 μL plasma or urine^57^. A total number of 13 Gabonese women were positive for *S. haematobium* by egg count and/or CAA in plasma (Supplemental Table 3). CAA was only detected in maternal plasma samples but not in cord blood.

30 women provided stool samples, which were prepared with standard methods for microscopic examination to determine the presence of intestinal helminth infections (Kato Katz, copro culture, McMaster, etc). Sample collection for parasitological examination was only performed at the time of delivery.

### Biochemical analysis

The concentrations of 25(OH)D using the Diasorin assay (Diasorin, Stillwater, MN, USA), as well as CRP and Calcium levels in all plasma samples were measured at the Institute for Clinical Chemistry and Pathobiochemistry at the Klinikum rechts der Isar of the Technical University of Munich according to the manufacturer’s specifications.

### Quantitative real-time Polymerase Chain Reaction (qRT-PCR)

Thawed fetal and maternal placenta samples were homogenized (using Precellys®24 tissue homogenizer) and RNA was isolated from using GenElute^™^ Mammalian Total RNA Miniprep Kit, according to manufacturer’s instructions. RNA yield and purity were measured using a NanoDrop® 1000 Spectrophotometer. The resulting RNA was stored at −80 °C until further processing. cDNA was prepared following the Promega usage information for first-strand cDNA synthesis with M-MLV RT (H-) Point Mutant and the resulting cDNA was stored at −20 °C until further processing. qRT-PCR was performed to measure the relative concentrations of certain genes, using Roche Universal Probe Library and HPRT as house-keeping gene. The LightCycler® Probes Master (Roche) is a ready-to-use reaction mix containing the Taq Polymerase and appropriate buffers. Gene sequences and probes are shown in Supplemental Table 2. The following qRT-PCR was performed in a Thermocycler C 1000 (Bio-Rad).

### Enzyme-linked immunosorbent assay (ELISA) for detection of total and AWA specific IgE and IgG4 levels

Plasma samples were transported on dry ice to Munich, Germany, for quantification of total IgE and IgG4 and to Leiden, the Netherlands, for AWA specific IgE and IgG4. Levels of total IgE and IgG4 were measured in all European and African maternal and cord plasma samples using the Invitrogen IgE, or IgG4 Human ELISA Kit, respectively, according to manufacturer’s instructions (Thermo Fischer). The plasma was 1:10 diluted for IgE measurement and 1:1000 for IgG4. Acquisition of the multiplex immunoassay was performed by using the LSRII flow cytometer (Becton Dickinson). *S. haematobium* adult worm antigen (AWA) specific IgG4 and IgE were measured by ELISA modified from previous protocols^58^. Briefly, maxisorp plates (Nunc, Roskilde, Denmark) were coated overnight with 5 mg/ml AWA diluted in carbonate buffer pH 9.6. For IgG4, the plates were blocked with PBS-5%BSA while for IgE with PBS-2%BSA. The plasma was diluted in PBS-0.05%Tween-5% FCS (Fetal calf serum) or Tris-HCl–0.05% Tween for IgG4 and IgE ELISA, respectively. The presence of IgG4 was shown by using HRP-labelled anti human IgG4 (1:3000) (Sanquin, Amsterdam, the Netherlands). For IgE assay, the detection antibodies used were biotinylated goat anti human IgE (1:1000; Vector Laboratories, Burlingame, CA, USA) followed by streptavidin HRP conjugate (1:10000; Sanquin, Amsterdam, the Netherlands). The assays were developed with tetramethylbenzidine (TMB) and stopped with 10% H2SO4. Absorbances were measured at 450 nm. The levels of antibody present in a given sample are expressed in arbitrary units (AU/ml) according to the standard curve of pooled positive *S. haematobium* plasma.

### Statistical analysis

Statistical analysis was performed by using SPSS for Windows version 23.0 (SPSS Inc., Chicago, IL) and PRISM® 5.01 (GraphPad Software Inc., San Diego, CA, USA). D’Agostino and Pearson omnibus normality tests were performed, and parametrically distributed data was analysed with unpaired T test (2 groups) and two-tailed Mann-Whitney-Test was used for non-parametric data. Statistical dependence was analysed by Spearman’s rank correlation coefficient. Categorical data were analysed by Pearson chi-squared test. Data are presented as median with interquartile range (IQR) or mean with standard deviation (SD).

In all cases, statistical significance was assumed with a p value < 0.05 and where significance level was reached, p values are indicated in individual graphs.

## Supplementary information

Supplementary figures

Supplementary tables

## Data Availability

All data generated or analysed during this study are included in this published article and its supplementary information files.
