# Author Correction: Placental gene expression and antibody levels of mother-neonate pairs reveal an enhanced risk for inflammation in a helminth endemic country

**DOI:** 10.1038/s41598-020-58618-y

**Published:** 2020-01-28

**Authors:** Esther Ludwig, Jutta Harder, Matthew Lacorcia, Yabo Josiane Honkpehedji, Odilon Paterne Nouatin, Govert J. van Dam, Paul L. A. M. Corstjens, Erliyani Sartono, Meral Esen, Silvia M. Lobmaier, Ayola Akim Adegnika, Clarissa Prazeres da Costa

**Affiliations:** 1 Institute for Medical Microbiology, Immunology and Hygiene, Technische Universität München, Munich, Germany; 2 Centre de Recherches Médicales de Lambaréné, Lambaréné, Gabon; 3 Department of Parasitology, Leiden University Medical Centre, Leiden, The Netherlands; 4 Institut für Tropenmedizin, Universität Tübingen, Tuebingen, Germany; 5 Frauenklinik und Poliklinik, Klinikum rechts der Isar, Technische Universität München, Munich, Germany; 6 German Centre for Infection Research, Tuebingen, Germany; 7 Department of Cell and Chemical Biology, Leiden University Medical Center, Leiden, The Netherlands

Correction to: *Scientific Reports* 10.1038/s41598-019-52074-z, published online 31 October 2019

The Acknowledgements section in this Article is incomplete.

“We are grateful to all pregnant women who participated in the study. We thank all CERMEL personnel for their exceptional assistance, and we are indebted to the midwives at the General Hospital and those at HAS, as well as the co-workers at CERMEL. Claudia de Dood (LUMC, Cell and Chemical Biology department) is acknowledged for preparing the UCP-LF CAA test materials and performing the CAA assays on the plasma and urine samples. This work was supported and funded by DZIF TI 07.003 and DFG CO 1469/8-1.”

should read:

“We are grateful to all pregnant women who participated in the study. We thank all CERMEL personnel for their exceptional assistance, and we are indebted to the midwives at the General Hospital and those at HAS, as well as the co-workers at CERMEL. Claudia de Dood (LUMC, Cell and Chemical Biology department) is acknowledged for preparing the UCP-LF CAA test materials and performing the CAA assays on the plasma and urine samples. This work was supported and funded by DZIF TI 07.003, DFG CO 1469/8-1 and DFG CO 1469/14-1.”

