# Supplementary material for: Placental gene expression and antibody levels of mother-neonate pairs reveal an enhanced risk for inflammation in a helminth endemic country
Source: Sci Rep. 2019 Oct 31;9:15776. doi: 10.1038/s41598-019-52074-z (PMC6823435; doi:10.1038/s41598-019-52074-z)
Supplement: Supplementary file 1 — Supplementary figures [file 41598_2019_52074_MOESM1_ESM.pdf]

Placental gene expression and antibody levels of mother-neonate pairs reveal an enhanced risk for inflammation in a helminth endemic country

AUTHORS:
Esther Ludwig[1], Jutta Harder[1], Matthew Lacorcia[1], Yabo Josiane Honkpehedji[2,3], Odilon Nouatin[2], Govert J. van Dam[3], Paul L.A.M. Corstjens[7], Erliyani Sartono[3], Meral Esen[4, 6], Silvia M. Lobmaier[5], Ayola Akim Adegnika,[2,3,4,6] and Clarissa Prazeres da Costa[1]*

ADDRESSES:

[1]Institute for Medical Microbiology, Immunology and Hygiene, Technische Universität München, Munich, Germany;

[2]Centre de Recherches Médicales de Lambaréné, Lambaréné, Gabon;

[3]Department of Parasitology, Leiden University Medical Centre, Leiden, The Netherlands.

[4]Institut für Tropenmedizin, Universität Tübingen, Tübingen, German;

[5]Frauenklinik und Poliklinik, Klinikum rechts der Isar, Technische Universität München, Munich, Germany;

[6]German Centre for Infection Research, Tuebingen, Germany

[7]Department of Cell and Chemical Biology, Leiden University Medical Center, Leiden, the Netherlands.

***CORRESPONDING AUTHOR:**

Prof. Dr. med. C. Prazeres da Costa

Institute for Medical Microbiology, Immunology and Hygiene

Technische Universität München (TUM)

Trogerstrasse 30, 81675 München, Germany.

Tel/Fax: 0049-89-4140-4130/-4868

Email: Clarissa.daCosta@tum.de

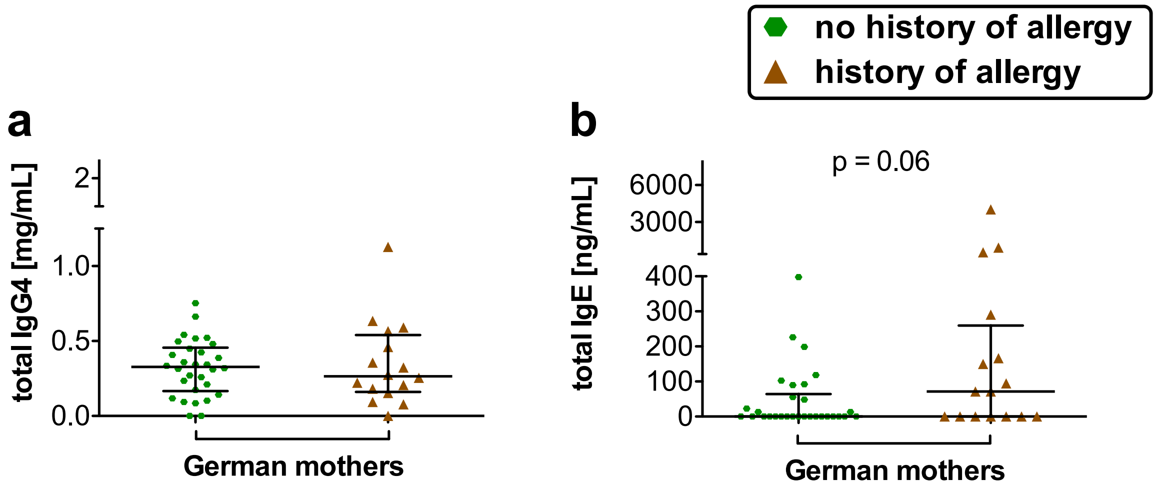

Supplementary figure 1: Total IgG4 and IgE levels in sera of German mothers with and without history of allergy.

Total IgG4 and IgE levels were measured in plasma via ELISA. All data are shown with median and interquartile range. P values are for Mann-Whitney U-tests. P value: * = < 0,05; ** = < 0,01; *** = < 0,001; **** = < 0,0001; n (maternal, Germany) = 47; n (cord, Germany) = 47; **(a)** total IgG4 levels in German mothers with and without history of allergy. **(b)** total IgE levels in German mothers with and without history of allergy.

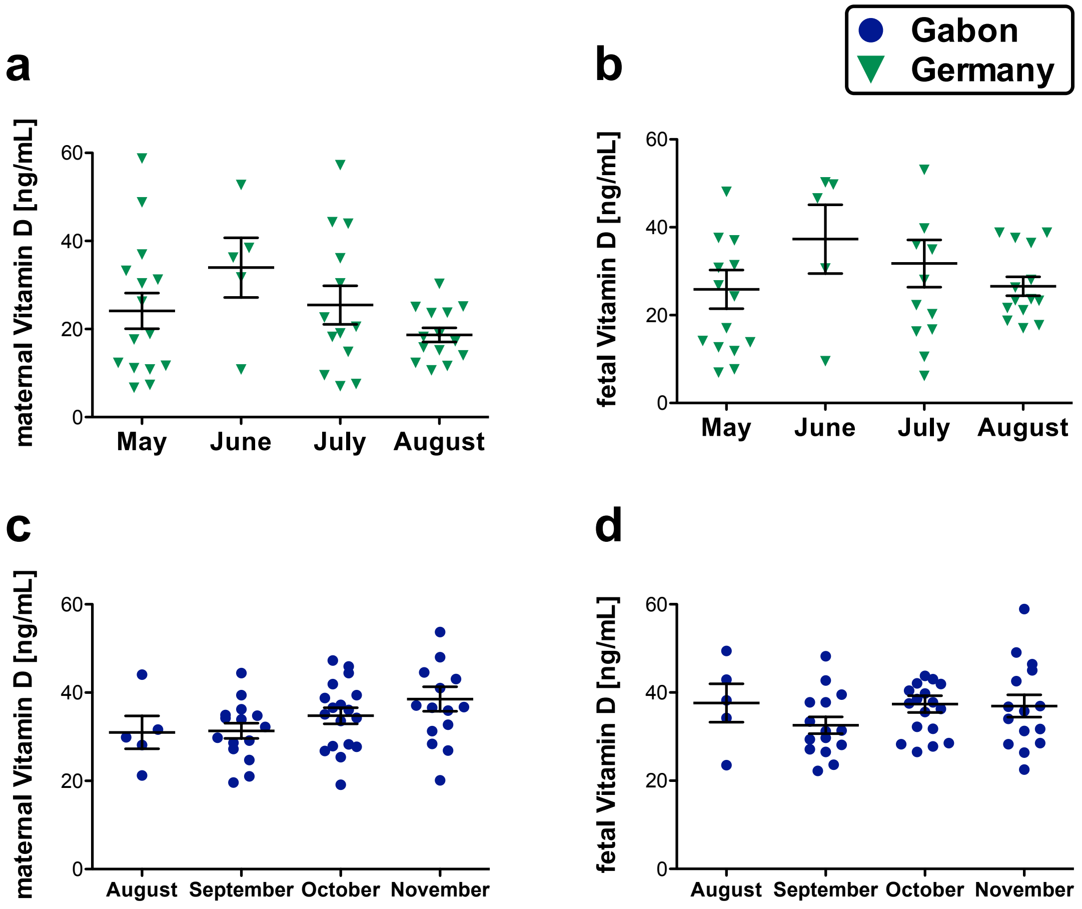

Supplementary figure 2: Association between 25(OH)D plasma levels and month of sample collection.

Plasma 25- (OH) vitamin D concentrations were measured using the Diasorin assay. All data are shown with mean and standard deviation. One-way ANOVA was used to investigate for significant differences between groups. . n (maternal, Gabon) = 53; n (maternal, Germany) = 47; n (cord, Gabon) = 52; n (cord, Germany) = 47; **(a)** German maternal 25(OH)D plasma levels divided into months of collection. **(b)** German fetal 25(OH)D plasma levels divided into months of collection. **(c)** Gabonese maternal 25(OH)D plasma levels divided into months of collection. **(d)** Gabonese fetal 25(OH)D plasma levels divided into months of collection.
