# Supplementary material for: Placental gene expression and antibody levels of mother-neonate pairs reveal an enhanced risk for inflammation in a helminth endemic country
Source: Sci Rep. 2019 Oct 31;9:15776. doi: 10.1038/s41598-019-52074-z (PMC6823435; doi:10.1038/s41598-019-52074-z)
Supplement: Supplementary file 2 — Supplementary tables [file 41598_2019_52074_MOESM2_ESM.pdf]

Placental gene expression and antibody levels of mother-neonate pairs reveal an enhanced risk for inflammation in a helminth endemic country

AUTHORS:
Esther Ludwig[1], Jutta Harder[1], Matthew Lacorcia[1], Yabo Josiane Honkpehedji[2,3], Odilon Nouatin[2], Govert J. van Dam[3], Paul L.A.M. Corstjens[7], Erliyani Sartono[3], Meral Esen[4, 6], Silvia M. Lobmaier[5], Ayola Akim Adegnika,[2,3,4,6] and Clarissa Prazeres da Costa[1]*

ADDRESSES:

[1]Institute for Medical Microbiology, Immunology and Hygiene, Technische Universität München, Munich, Germany;

[2]Centre de Recherches Médicales de Lambaréné, Lambaréné, Gabon;

[3]Department of Parasitology, Leiden University Medical Centre, Leiden, The Netherlands.

[4]Institut für Tropenmedizin, Universität Tübingen, Tübingen, German;

[5]Frauenklinik und Poliklinik, Klinikum rechts der Isar, Technische Universität München, Munich, Germany;

[6]German Centre for Infection Research, Tuebingen, Germany

[7]Department of Cell and Chemical Biology, Leiden University Medical Center, Leiden, the Netherlands.

***CORRESPONDING AUTHOR:**

Prof. Dr. med. C. Prazeres da Costa

Institute for Medical Microbiology, Immunology and Hygiene

Technische Universität München (TUM)

Trogerstrasse 30, 81675 München, Germany.

Tel/Fax: 0049-89-4140-4130/-4868

Email: Clarissa.daCosta@tum.de

	Gabon		p value
	Negative for *Schistosoma haematobium*	Positive for *Schistosoma haematobium*	
**Number n**	41	13	
**Mother**			
Age [years]	26 ± 6	27 ± 5	0.66
Parity	2 ± 2	2 ± 2	0.84
Gravidity	4 ± 2.1	4 ± 1.6	0.69
Gestational age at delivery (weeks)	39.4 ± 2	39.0 ± 2	0.66
Haemoglobin [g/dL]	10.64 ± 2.36	10.42 ± 1.71	0.54
White blood cells [10^3/mm^3]	11.77 ± 5.47	11.94 ± 4.15	0.92
Eosinophils [%]	1.74 ± 1.86	2.40 ± 2.03	0.14
25-OHD-Vitamin D3 concentration [ng/mL]	33.4 ± 8.7	37.8 ± 8.9	0.19
Calcium [mmol/L]	2.29 ± 0.12	2.26 ± 0.11	0.35
CRP [mg/dL]	1.05 ± [0.66; 2.25]	1.43 ± [0.28; 2.01]	0.76
**Newborn**			
Gender (male)	24 (59)	6 (46)	0.27
Length [cm]	50 ± 2.7	50 ± 2.2	0.56
Birthweight [g]	3054 ± 535	3145 ± 436	0.58
25-OHD-Vitamin D3 concentration [ng/mL]	34.8 ± 7.9	39.6 ± 10.0	0.08
Calcium [mmol/L]	2.68 ± 0.15	2.61 ± 0.37	0.92
CRP [mg/dL]	0.01 ± 0.01	0.01 ± 0.01	0.50

Supplementary table 1: Characteristics of S. haematobium infected and non-infected mothers and their newborns at delivery in Gabon.

Data are presented as mean ± SD (n), median with IQR (n) for C-reactive Protein (CRP), values or as numbers (%) where indicated; T-test was performed where data are normal distributed; for data without normal distribution a two-tailed Mann Whitney U-test was performed; blood for plasma parameters was taken from maternal peripheral vein blood or from cord blood, respectively; *S. haematobium* diagnostics was done by egg count or detection of levels of schistosome specific circulating anodic antigen (CAA) in plasma samples.

**HPRT (reference gene)**	
forward primer	5' tgaccttgatttattttgcatacc 3'
reverse primer	5' cgagcaagacgttcagtcct 3'
dual labelled probe	Universal ProbeLibrary Probe #73, Roche
**VDR 1**	
forward primer	5’ gaagctgaacttgcatgagga 3’
reverse primer	5’ gtcctggatggcctcaatc 3’
dual labelled probe	Universal ProbeLibrary Probe #15, Roche
**Cyp27b1**	TTGGCAAGCGCAGCTGTAT
forward primer	5' cgcagctgtatggggaga 3'
reverse primer	5'cacctcaaaatgtgttaggatctg 3'
dual labelled probe	Universal ProbeLibrary Probe #53, Roche
**Foxp3**	
forward primer	5' cttccttgaaccccatgc 3'
reverse primer	5' gagggtgccaccatgacta 3'
dual labelled probe	Universal ProbeLibrary Probe #44, Roche
**Hsd3b1**	
forward primer	5' tcttcggtgtcactcacagag 3'
reverse primer	5' ggcacactagcttggacaca 3'
dual labelled probe	Universal ProbeLibrary Probe #17, Roche
IL10	
forward primer	5' gatgccttcagcagagtgaa 3'
reverse primer	5' gcaacccaggtaacccttaaa 3'
dual labelled probe	Universal ProbeLibrary Probe #67, Roche
IFNG	
forward primer	5' ggcattttgaagaattggaaag 3'
reverse primer	5' tttggatgctctggtcatctt 3'
dual labelled probe	Universal ProbeLibrary Probe #21, Roche

Supplementary table 2: Primer sequences.

Primer sequences used for the qtPCR.

ID	R=T/FC	pg/mL	Egg count positive
M001	0,020	1	no
F001	0,024	2	
M002	1,691	**832**	**yes**
F002	0,030	3	
M003	0,019	1	no
F003	0,038	4	
M004	0,028	2	no
F004	0,000	0	
M006	0,026	2	no
F006	0,023	2	
M007	0,022	2	no
F007	0,038	4	
M008	0,155	**30**	**yes**
F008	0,021	1	
M009	0,022	2	no
F009	0,009	0	
M010	0,021	1	no
F010	0,019	1	
M011	0,042	5	no
F011	0,024	2	
M012	0,032	3	no
F012	0,017	1	
M014	0,019	1	no
F014	0,023	2	
M015	0,037	4	no
F015	0,025	2	
M013	0,026	2	no
F013	0,018	1	
M016	0,048	6	**yes**
F016	0,029	3	
M017	0,044	5	no
F017	0,020	1	
M018	0,058	8	no
F018	0,000	0	
M020	0,014	1	no
F020	0,020	1	
M021	1,519	**704**	no
F021	0,038	4	
M022	0,640	**203**	**yes**

F022	0,021	2	
M026	0,126	**23**	no
F026	0,024	2	
M025	0,018	1	no
F025	0,038	4	
M027	0,024	2	no
F027	0,022	2	
M028	0,033	3	no
F028	0,024	2	
M029	0,051	7	no
F029	0,030	3	
M030	0,028	3	no
F030	0,039	4	
M031	0,012	0	no
F031	0,023	2	
M032	0,025	2	no
F032	0,043	5	
M033	0,024	2	no
F033	0,028	3	
M034	0,041	5	no
F034	0,030	3	
M036	0,255	**59**	**yes**
F036	0,048	6	
M037	0,028	2	no
F037	0,044	5	
M038	0,030	3	no
F038	0,022	2	
M039	0,370	**97**	**yes**
F039	0,020	1	
M040	0,035	4	no
F040	0,037	4	
M041	0,242	**55**	**yes**
F041	0,026	2	
M042	0,024	2	no
F042	0,023	2	
M043	0,031	3	no
F043	0,027	2	
M044	0,024	2	no
F044	0,031	3	
M045	0,022	2	no

F045	0,025	2	
M046	0,249	**57**	no
F046	0,044	5	
M047	0,026	2	no
F047	0,020	1	
M048	2,793	**1966**	no
F048	0,017	1	
M049	0,027	2	no
F049	0,019	1	
M050	0,021	2	no
F050	0,026	2	
M051	0,045	5	no
F051	0,027	2	
M052	0,029	3	no
F052	0,015	1	
M053	0,025	2	no
F053	0,021	1	
M057	0,076	**11**	**yes**
F057	0,019	1	
M054	0,025	2	no
M055	0,018	1	no
F055	0,013	1	
M056	0,013	0	no
F056	0,018	1	
M071	3,129	**2454**	no
F071	0,021	1	
M072	0,706	**233**	no
F072	0,015	1	

Supplementary table 3: CAA diagnostics and egg count in urine of Gabonese women. Levels of schistosome circulating antigen (circulating anodic antigen [CAA]) were measured in cord and maternal peripheral plasma as well as in urine utilizing an immunochromatography based assay Egg count was done via microscopy in one to three urine samples from Gabonese mothers given at time of delivery and within a few days afterwards. Maternal samples are indicated with “M” and cord blood samples with “F”.
